# Supplementary material for: Acupuncture (Jin’s Three-Needle) versus sham acupuncture in treating mild-to-moderate depression: Study protocol for randomized clinical trial
Source: PLoS One. 2025 Dec 5;20(12):e0338341. doi: 10.1371/journal.pone.0338341 (PMC12680233; doi:10.1371/journal.pone.0338341)
Supplement: S1 File — (PDF) [file pone.0338341.s001.pdf]

Acupuncture (Jin's Three-Needle) versus sham acupuncture in Treating Mild-to-Moderate Depression: Study Protocol for Randomized Clinical Trial

Supplementary file

Contents

S1 SPIRIT 2025 checklist of items to address in a randomised trial protocol* 2
S2 Standards for Reporting Interventions in Clinical Trials of Acupuncture(STRICTA) 5
S3 Sham Acupoint Locations 7
S4 Molecular analysis details 8

S1 SPIRIT 2025 checklist of items to address in a randomised trial protocol*

Section/topic	No	SPIRIT 2025 checklist item description	Addressed on Page
**Administrative information**			
Title and structured summary	1a	Title stating the trial design, population, and interventions, with identification as a protocol	1−2
	1b	Structured summary of trial design and methods, including items from the World Health Organization Trial Registration Data Set	2
Protocol version	2	Version date and identifier	18
Roles and responsibilities	3a	Names, affiliations, and roles of protocol contributors	18−19
	3b	Name and contact information for the trial sponsor	18
	3c	Role of trial sponsor and funders in design, conduct, analysis, and reporting of trial; including any authority over these activities	18
	3d	Composition, roles, and responsibilities of the coordinating site,steering committee, endpoint adjudication committee, data management team, and other individuals or groups overseeing the trial, if applicable	NA
**Open science**			
Trial registration	4	Name of trial registry, identifying number (with URL.), and date of registration. If not yet registered, name of intended registry	1
Protocol and statistical analysis plan	5	Where the trial protocol and statistical analysis plan can be accessed will be accessible	19
Data sharing	6	Where and how the individual de-identified participant data(including data dictionary), statistical code, and any other materials	19
Funding and conflicts of interest	7a	Sources of funding and other support (eg, supply of drugs)	18
	7b	Financial and other conflicts of interest for principal investigators and steering committee members	18
Dissemination policy	8	Puns to communicate trial results to participants, health care professionals, the public, and other relevant groups (eg, reporting in trial registry, plain language summary, publication)	5
Introduction			
Background and rationale	9a	Scientific background and rationale, including summary of relevant studies (published and unpublished) examining benefits and harms for each intervention	3-4
	9b	Explanation for choice of comparator	4
Objectives	10	Specific objectives related to benefits and harms	4
**Methods: Patient and public involvement, trial design**			
Patient and public involvement	11	Details of, or plans for, patient or public involvement in the design,conduct, and reporting of the trial	4
Trial design	12	Description of trial design including type of trial (cg, parallel group,crossover), allocation ratio, and framework (eg, superiority,equivalence, non-inferiority, exploratory)	4

**Methods: Participants, interventions, and outcomes**			
Trial setting	13	Settings (eg, community, hospital) and locations (eg, countries, sites)where the trial will be conducted	5
Eligibility criteria	14a	Eligibility criteria for participants	
	14b	If applicable, eligibility criteria for sites and for individuals who will deliver the interventions (eg, surgeons, physiotherapists)	5-6
Intervention and comparator	15a	Intervention and comparator with sufficient details to allow replication including how, when, and by whom they will be administered. If relevant, where additional materials describing the intervention and comparator (eg, intervention manual) can be accessed	7-9
	15b	Criteria for discontinuing or modifying allocated intervention/comparator for a trial participant (eg, drug dose change in response to harms, participant request, or improving/worsening disease)	14
	15c	Strategies to improve adherence to intervention/comparator protocols, if applicable, and any procedures for monitoring adherence(eg, drug tablet return, sessions attended)	14
	15d	Concomitant care that is permitted or permitted during the trial	14
Outcomes	16	Primary and secondary outcomes, including the specific measurement variable (eg, systolic blood pressure), analysis metric (eg, change from baseline, final value, time to event), method of aggregation (og,median, proportion), and time point for each outcome	11-13
Harms	17	How harms are defined and will be assessed (eg, systematically, non-systematically)	13-14
Participant timeline	18	Time schedule of enrolment, interventions (including any run-ins and washouts), assessments, and visits for participants. A schematic diagram is highly recommended	8
Sample size	19	How sample size was determined, including all assumptions supporting the sample size calculation	14-15
Recruitment	20	Strategies for achieving adequate participant enrolment to reach target sample size	5-6
**Methods: Assignment of interventions**			
Randomisation:			
Sequence generation	21a	Who will generate the random allocation sequence and the method used	6
	21b	Type of randomisation (simple or restricted) and details of any factors for stratification. To reduce predictability of a random sequence, other details of any planned restriction (eg, blocking) should be provided in a separate document that is unavailable to those who enrol participants or assign interventions	6
Allocation concealment mechanism	22	Mechanism used to implement the random allocation sequence (eg,central computer/telephone: sequentially numbered, opaque, sealed containers), describing any steps to conceal the sequence until interventions are assigned	6
Implementation	23	Whether the personnel who will enrol and those who will assign participants to the interventions will have access to the random allocation sequence	6
Blinding	24a	Who will be blinded after assignment to interventions (eg,participants, care providers, outcome assessors, data analysts)	7
	24b	If blinded, how blinding will be achieved and description of the similarity of interventions	7
	24c	If blinded, circumstances under which unblinding is permissible, and procedure for revealing a participant's allocated intervention during the trial	7

Methods: Data collection, management, and analysis			
Data collection methods	25a	Plans for assessment and collection of trial data, including any related processes to promote data quality (eg, duplicate measurements,training of assessors) and a description of trial instruments (eg,questionnaires, laboratory tests) along with their reliability and accessed. if not in the protocol	14
	25b	Plans to promote participant retention and complete follow-up,including list of any outcome data to be collected for participants who discontinue or deviate from intervention protocols	14
Data management	26	Plans for data entry, coding, security, and storage, including any related processes to promote data quality (eg double data entry; range checks for data values). Reference to where details of data management procedures can be accessed, if not in the protocol	14
Statistical methods	27a	Statistical methods used to compare groups for primary and secondary outcomes, including harms	15-16
	27b	Definition of who will be included in each analysis (eg, all randomised participants), and in which group	15-16
	27c	How missing data will be handled in the analysis	15-16
	27d	Methods for any additional analyse (eg, subgroup and sensitivity analyses)	15-16
**Methods: Monitoring**			
Data monitoring committee	28a	Composition of data monitoring committee (DMC); summary of its role and reporting structure; statement of whether it is independent from the sponsor and funder; conflicts of interest and reference to where further details about its charter can be found, if not in the protocol.Alternatively, an explanation of why a DMC is not needed	14
	28b	Explanation of any interim analyses and stopping guidelines, including who will have access to these interim results and make the final decision to terminate the trial	14
Trial monitoring	29	Frequency and procedures for monitoring trial conduct. If there is no monitoring, give explanation	14
**Ethics**			
Research ethics approval	30	Plans for seeking research ethics committee/institutional review board approval	5
Protocol amendments	31	Plans for communicating important protocol modifications to relevant parties	5
Consent or assent	32a	Who will obtain informed consent or assent from potential trial participants or authorised proxies, and how	5-6
	32b	Additional consent provisions for collection and use of participant data and biological specimens in ancillary studies, if applicable	5-6
Confidentiality	33	How personal information about potential and enrolled participants will be collected, shared, and maintained in order to protect confidentiality before, during, and after the trial	5-6
Ancillary and post-trial care	34	Provisions if any, for ancillary and post-trial care, and for compensation to those who suffer harm from trial participation	NA

*Hróbjartsson A, Boutron I, Hopewell S, Moher D, Schulz KF, Collins GS, Tunn R, Aggarwal R, Berkwits M, Berlin JA, Bhandari N, Butcher NJ, Campbell MK, Chidebe RCW, Elbourne DR, Farmer AJ, Fergusson DA, Golub RM, Goodman SN, Hoffmann TC, Ioannidis JPA, Kahan BC, Knowles RL, Lamb SE, Lewis S, Loder E, Offringa M, Ravaud P, Richards DP, Rockhold FW, Schriger DL, Siegfried NL, Staniszewska S, Taylor RS, Thabane L, Torgerson DJ, Vohra S, White IR, Chan AW. SPIRIT 2025 explanation and elaboration: updated guideline for protocols of randomised trials. BMJ. 2025 Apr 28;389:e081660. doi: 10.1136/bmj-2024-081660. PMID:

40294956; PMCID: PMC12128891.

S2 Standards for Reporting Interventions in Clinical Trials of Acupuncture(STRICTA)

Section/topic	Item number	Checklist item	Report/ Not
1.Acupuncture rationale			
	1a	Style of acupuncture (eg, Traditional Chinese Medicine, Japanese, Korean, Western medical, Five Element, ear acupuncture, etc).	P3-4
	1b	Reasoning for treatment provided, based on historical context, literature sources and/or consensus methods, with references where appropriate.	P3-4
	1c	Extent to which treatment was varied.	P7-11
2. Details of needling			
	2a	Number of needle insertions per subject per session (mean and range where relevant)	Table 1
	2b	Names (or location if no standard name) of points used (uni-/bilateral).	Table 1
	2c	Depth of insertion, based on a specified unit of measurement or on a particular tissue level.	P9
	2d	Responses sought (eg, *de qi* or muscle twitch response).	P9
	2e	Needle stimulation (eg, manual or electrical).	P9
	2f	Needle retention time.	P9
	2g	Needle type (diameter, length and manufacturer or material).	P9
3. Treatment regimen			
	3a	Number of treatment sessions.	P9
	3b	Frequency and duration of treatment sessions.	P9

4. Other components of treatment			
	4a	Details of other interventions administered to the acupuncture group (eg, moxibustion, cupping, herbs, exercises, lifestyle advice).	NA
	4b	Setting and context of treatment, including instructions to practitioners, and information and explanations to patients.	P7
5. Practitioner background			
	5	Description of participating acupuncturists (qualification or professional affiliation, years in acupuncture practice, other relevant experience).	P7
6. Control or comparator interventions			
	6a	Rationale for the control or comparator in the context of the research question, with sources that justify the choice(s).	P7-8
	6b	Precise description of the control or comparator. If sham acupuncture or any other type of acupuncture-like control is used, provide details as for items 1-3 above.	

This checklist should be read in conjunction with the explanations of the Standards for Reporting Interventions in Clinical Trials of Acupuncture items.

S3 Sham Acupoint Locations

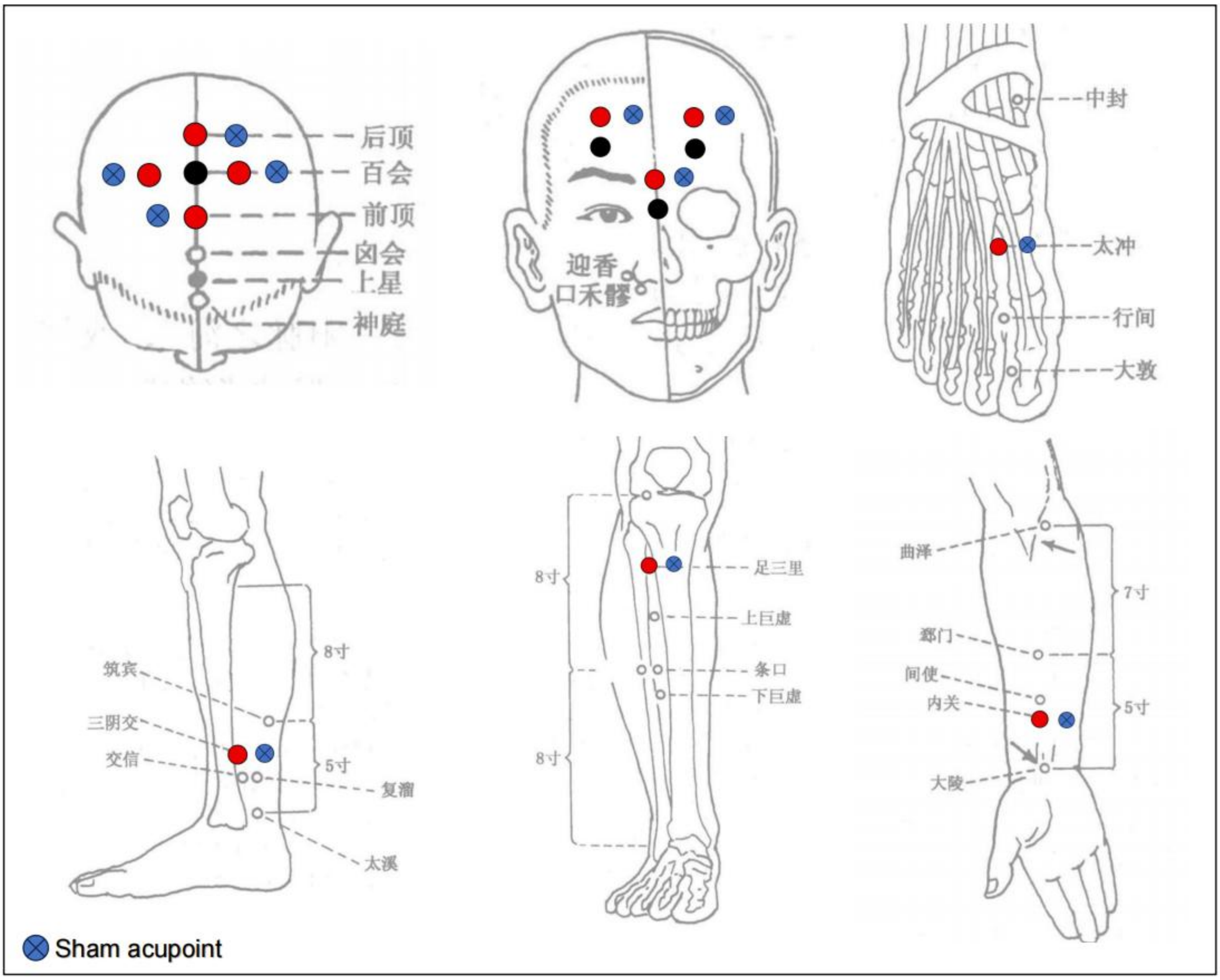

S4 Molecular analysis details

Collection of patient blood samples

Whole blood will be collected from 8 patients before and after intervention, and from 10 healthy volunteers. Draw 10ml of venous blood on an EDTA anticoagulant tube on an empty stomach. Let it stand at room temperature for 1 hour. Then, centrifuge at 3000rpm for 10 minutes at 4°C to obtain the supernatant plasma sample. DNA will be isolated from the peripheral blood leukocytes using QIAamp DNA mini kit (Qiagen) . The isolated DNA will be stored at - 80° C, with a temperature monitoring system in place to maintain the required storage conditions.

Data quality control and standardization

The Illumina iScan system scans the chip to generate ldat files. These ldat files are imported into R, and the ChAMP package will be used for preprocessing to obtain the raw data. The raw data (idat files) will be normalized using the BMIQ method. Signal values (Beta values) representing DNA methylation levels at CpG sites will be then extracted. Beta values (β) range from 0 to1, indicating the methylation level at a given site. Values closer to 0 suggest lower methylation, while values closer to 1 indicate higher methylation. The calculation formula for the Beta value is as follows:

$$\mathbf{Beta_i=M_i/(M_i+U_i+offset)}$$

"M" represents the methylation signal intensity, "U" represents the non-methylation signal intensity, and "offset" is an adjustment value used to prevent the denominator from being zero.

DNA methylation analysis

The MethylationEPIC BeadChip experiment and data analysis of all samples will be conducted by OE Biotechnology Co. Ltd. (Shanghai, China). DNA concentration and integrity will be evaluated by NanoDrop 2000 spectrophotometer (Thermo Fisher Scientific, Waltham, MA, USA) and agarose gel electrophoresis respectively. DNA will be treated with bisulfite using the Zymo Research EZ DNA methylation-Glod Kit

(Zymo Research, Irvine, CA, USA), and analyzed on the Illumina Infinium MethylationEPIC (850 K) BeadChip (Illumina). The chips will be scanned with Illumina iSCAN, and the Idat files will be imported and preprocessed with ChAMP (version 2.12.4) package in R to obtain the raw data. The BMIQ method will be used to normalize the raw data.
