# Supplementary material for: Acupuncture (Jin’s Three-Needle) versus sham acupuncture in treating mild-to-moderate depression: Study protocol for randomized clinical trial
Source: PLoS One. 2025 Dec 5;20(12):e0338341. doi: 10.1371/journal.pone.0338341 (PMC12680233; doi:10.1371/journal.pone.0338341)
Supplement: Attachment — Submitted filename: Response Letter.docx [file pone.0338341.s002.docx]

Dear Editor,

We would like to resubmit the revised manuscript entitled “Acupuncture (Jin's Three-Needle) versus sham acupuncture in Treating Mild-to-Moderate Depression: Study Protocol for Randomized Clinical Trial” (Manuscript NO: PONE-D-25-44858) for your consideration. We would like to thank the reviewers for thoroughly and patiently reviewing our manuscript and making many thoughtful comments.

After we have carefully read the comments from the reviewer, point-by-point responses to the reviewers’ comments are enclosed after the letter for your consideration.

Look forward to hearing from you soon,

Yours sincerely,

Hao Wen (PhD)

1.Guangzhou Huangpu District Hospital of Traditional Chinese Medicine

2.School of Traditional Chinese Medicine, Jinan University

Tel: 86+ 15876576899

Email: Wenhaophd@outlook.com

### Response letter for manuscript “Acupuncture (Jin's Three-Needle) versus sham acupuncture in Treating Mild-to-Moderate Depression: Study Protocol for Randomized Clinical Trial”

We thank the Editor and reviewers for their constructive comments and suggestions regarding this manuscript. We have revised the manuscript accordingly, and provided the point-by-point responses below. Each comment is highlighted in bold font, and our response follows each comment. We thank the editorial team for reviewing the revised manuscript.

**Journal Requirements:**

**When submitting your revision, we need you to address these additional requirements.**

**1. Please ensure that your manuscript meets PLOS ONE's style requirements, including those for file naming.**

Following your suggestion, we have reviewed the manuscript and revised it.

**2. We noticed you have some minor occurrence of overlapping text with the following previous publication(s), which needs to be addressed:**

**https://doi.org/10.1016/j.heliyon.2024.e28889**

**https://doi.org/10.3389/fpsyt.2025.1624825**

**In your revision ensure you cite all your sources (including your own works), and quote or rephrase any duplicated text outside the methods section. Further consideration is dependent on these concerns being addressed.**

Following your suggestion, we have revised it.

**3. Your ethics statement should only appear in the Methods section of your manuscript. If your ethics statement is written in any section besides the Methods, please delete it from any other section.**

Following your suggestion, we have revised it.

**4. We note that Figure 3 in your submission contain copyrighted images. All PLOS content is published under the Creative Commons Attribution License (CC BY 4.0), which means that the manuscript, images, and Supporting Information files will be freely available online, and any third party is permitted to access, download, copy, distribute, and use these materials in any way, even commercially, with proper attribution.**

Thank you for bringing this important issue to our attention. We fully acknowledge the requirements of the Creative Commons Attribution License (CC BY 4.0) for PLOS publications and apologize for the oversight regarding copyrighted material in Figure 3.

As we were unable to secure authorization from the copyright holders of the original images, we have replaced all copyrighted figures in the manuscript with original, hand-drawn illustrations of the acupoints. These new diagrams are created by our team, ensuring full compliance with copyright regulations and the CC BY 4.0 license. The revised Figure 3 now features these non-copyrighted, schematic representations, which maintain scientific accuracy while respecting intellectual property rights.

**5. Please include captions for your Supporting Information files** **at the end of your manuscript, and update any in-text citations to match accordingly**

Following your suggestion, we have added “Supporting Information” at the end of the manuscript.

**Reviewer #1:**

**The manuscript needs a good editor for missing articles, etc.**

**There are some statistical aspects that need clarification on sample size and analysis.**

**There is no clinically relevant treatment effect given. The study should be powered on a clinically relevant difference, not the difference found in a prior study.**

Thank you very much for your insightful comments and valuable feedback on the statistical aspects of sample size and analysis in our study. We fully understand your concern regarding the lack of a specified clinically relevant treatment effect and the need to power the study on a clinically relevant difference rather than the difference found in a prior study.

We acknowledge that ideally, the sample size calculation should be based on a well-established minimum clinically important difference (MCID) for the HAMD - 17 scale. However, as you are aware, currently, there is no universally agreed - upon and fixed MCID value for the HAMD - 17. Different studies have employed various methods to estimate the MCID, and the results have shown variations.

The HAMD - 17 is primarily used to assess the severity of depressive symptoms and evaluate treatment effects. The change in its total score can reflect the evolution of the disease condition. But determining a specific MCID value requires consideration of the specific research background and the estimation method used.

In our study, we referred to the difference found in a previous study as a preliminary reference for sample size calculation. We understand that this may not fully represent a clinically relevant difference. To address this issue, we plan to conduct a sensitivity analysis after the study is completed. This analysis will assess how different assumed MCID values (within a reasonable range based on existing literature and expert opinions) would affect the study's results and conclusions.

Moreover, during the study design phase, we consulted with several experts in the field of depression research and clinical practice. They suggested that, given the current lack of a definite MCID for the HAMD - 17, using the data from a relevant prior study as a starting point for sample size estimation is a practical approach. At the same time, we will ensure that the study has sufficient power to detect meaningful differences in treatment effects, even if these differences do not precisely match a pre - defined MCID.

We appreciate your attention to this important aspect of our study, and we are committed to ensuring that our research is methodologically sound and clinically relevant. We will continue to monitor the development of MCID research for the HAMD - 17 and incorporate any new findings into our future work.

We will clearly state these planned analyses in our updated statistical analysis plan.

**It is likely that HAMD scores are not normally distributed, and the analysis will be a repeated measures ANOVA. What distributional assumptions were made in the sample size computation?**

You are correct in pointing out that HAMD scores may not follow a normal distribution. In our sample size computation using PASS11.0 software, the underlying assumption was based on the data from the previous study, which provided the mean and standard deviation values for the HAMD - 17 score differences between the baseline and the endpoint in both the acupuncture and sham acupuncture groups.

The software, by default, makes certain distributional assumptions for the calculations. However, it is important to note that PASS11.0 uses algorithms that are robust to moderate departures from normality, especially when dealing with large enough sample sizes as we have planned (45 subjects per group before accounting for drop - outs).

In practice, for repeated measures ANOVA, which we intend to use for analyzing outcomes with multiple time points, the assumption of normality of the residuals is more critical. We plan to conduct normality tests (such as the Shapiro - Wilk test) on the residuals after fitting the repeated measures ANOVA model. If the normality assumption is violated, we will consider appropriate non - parametric alternatives, such as the generalized estimating equations (GEE) approach with a suitable link function or non - parametric repeated measures methods.

We will clearly state these planned analyses in our updated statistical analysis plan.

**the type I error RATE (word missing) is set at 0.05. Two sided?**

Yes, the type I error rate is set at 0.05, and it is a two - sided test. This means that we are interested in detecting any significant difference (either an increase or a decrease) in the HAMD - 17 scores between the acupuncture and sham acupuncture groups. A two - sided test is appropriate in this context as we do not have a priori knowledge about the direction of the treatment effect, and we want to be able to identify both positive and negative differences that are statistically significant.

**No loss to follow-up adjustment?**

We did make an adjustment for loss to follow - up in our sample size calculation. As mentioned in the original description, we considered a dropout rate of 15%. Based on this rate, we increased the initial sample size per group (calculated to be 45 subjects) to 53 subjects per group, resulting in a total of 106 subjects for the study with a 1:1 allocation ratio between the two groups.

This adjustment is crucial to ensure that we have sufficient power to detect statistically significant differences even if some subjects drop out during the course of the study. By accounting for potential dropouts, we aim to maintain the integrity of the study results and minimize the impact of missing data on the statistical analysis.

**I don't understand how the study statistician can be "unaware of the study". How can someone unaware of the study analyze the study? Are you saying blinded? Why would the statistician need to be blinded? How can you analyze a treatment effect if you don't know the treatments?**

We sincerely apologize for the confusion caused by our imprecise language. The statistician is, of course, fully aware of the study's design, hypotheses, and analysis plan. The key point we failed to articulate clearly is that the statistician is blinded to the treatment group allocation during the statistical analysis.

Let us clarify the process:

1. What the Statistician Knows: The study statistician is fully briefed on the trial protocol, including the fact that there are two groups (Group A and Group B), the primary and secondary outcomes, the statistical models to be used (e.g., repeated-measures ANOVA), and the definition of the datasets (FAS, PPS, SS).

2. What the Statistician Does Not Know (The Blinding): Throughout the analysis phase, the two study groups are labeled with a non-revealing, neutral code (e.g., "Group 1" and "Group 2"). The statistician is not told which code corresponds to the 'JTN group' and which corresponds to the 'Sham Acupuncture group'. This is the state of being "blinded."

3. Why This Blinding is Crucial: Blinding the statistician is a best practice in high-quality clinical trials to prevent unconscious bias during the data analysis. If the statistician knows which group is which, there is a potential, however unintentional, to:

a. Make different choices in handling outliers or missing data for one group over the other.

b. Run additional, unplanned subgroup analyses selectively for one group.

c. Interpret marginal results (p-values just above or below 0.05) with a bias toward the expected outcome.

By remaining blinded, the statistician conducts the analysis exactly as pre-specified in the statistical analysis plan, ensuring the results are objective and unbiased.

4. How the Analysis is Performed and Interpreted: The statistician runs all the pre-planned analyses on the datasets labeled as "Group 1" and "Group 2." They produce the final output—tables, figures, and p-values—comparing "Group 1 vs. Group 2." Once this blinded analysis is complete and locked, the blinding is broken. Only then is the simple code revealed (e.g., "Group 1 = JTN, Group 2 = Sham"), and the results are interpreted accordingly in the manuscript.

In summary, the statistician is not "unaware of the study" but is "blinded to the treatment identity" during the data analysis phase. This is a rigorous safeguard to ensure the integrity of the statistical results. We will correct this inaccurate phrasing in our manuscript to clearly state: "The final statistical analyses will be conducted by an independent data analyst who is blinded to the treatment group allocation. " We thank the reviewer for prompting this essential clarification.

**The protocol mentions normality and non-normality in the repeated measures analyses, but there are no diagnostic or goodness of fit techniques mentioned.**

It is true that our initial protocol did not explicitly outline the specific diagnostic or goodness - of - fit techniques for assessing normality and non - normality in the repeated measures analyses. This was an oversight on our part, and we are grateful for your bringing it to our attention.

We plan to use the Shapiro - Wilk test to assess the normality of the residuals in the repeated measures ANOVA model. This test is known for its high power in detecting non - normality, especially for small to moderate sample sizes. For each time point and for the overall residuals of the model, we will calculate the Shapiro - Wilk statistic and the corresponding p - value. If the p - value is less than the pre - specified significance level (e.g., 0.05), we will reject the null hypothesis of normality.

Goodness - of - Fit Techniques for Non - Normal Data:

1. Generalized Estimating Equations (GEE): If the normality assumption is violated, we will consider using generalized estimating equations. GEE is a flexible approach for analyzing repeated measures data, especially when the response variable does not follow a normal distribution. It allows us to specify different link functions (e.g., logit, probit, identity) and correlation structures (e.g., exchangeable, autoregressive) depending on the nature of the data. We will select the appropriate link function and correlation structure based on the characteristics of our HAMD - 17 score data.

2. Non - Parametric Tests: Another option for non - normal data is to use non - parametric tests for repeated measures. For example, the Friedman test can be used to compare the distributions of the HAMD - 17 scores across multiple time points within each group. If significant differences are found, post - hoc pairwise comparisons can be made using non - parametric methods such as the Wilcoxon signed - rank test.

We will incorporate these diagnostic and goodness - of - fit techniques into our statistical analysis plan. Before conducting the main repeated measures analyses, we will first perform the normality tests. Based on the results, we will decide whether to proceed with the standard repeated measures ANOVA or use one of the alternative methods for non - normal data.

**Reviewer #2:**

**This article provides a new acupuncture treatment approach for early to mid-stage depression. I have two suggestions for the authors' consideration.**

**Could you please attach a reference picture of the sham acupuncture? This would allow readers to more intuitively understand how the sham acupuncture procedure was performed.**

Thank you for your valuable suggestion to enhance the clarity of our sham acupuncture procedure. We appreciate your emphasis on providing intuitive visual aids for readers. Following your recommendation, we have now included a detailed schematic diagram of the sham acupoint locations in Supplementary Material S3. This figure illustrates the precise positioning of the sham acupoints (located 1 inch lateral to the true acupoints, in non-meridian, non-acupoint regions) relative to the anatomical landmarks and true acupoints.

**Previous clinical or experimental studies on acupuncture for depression exist. Please discuss in the manuscript how the Jin's Three Needle technique differs from and what advantages it may have over other acupuncture approaches studied.**

Thank you for your constructive suggestion to elaborate on the distinctiveness and potential advantages of the Jin’s Three-Needle (JTN) technique compared to other acupuncture approaches for depression. We have revised the Discussion section to address this point comprehensively, as outlined below:

*Unlike conventional acupuncture, JTN integrates local, influential, and empirical points to achieve layered therapeutic effects on somatic, emotional, and energetic levels. For instance, the combination of HT7 (Shenmen), ST36 (Zusanli), and GB20 (Fengchi) in JTN simultaneously targets serotonin regulation, immune modulation, and vagal nerve activation [36]* *Lin, W., et al. suggest JTN may offer superior symptom relief and lower tolerance risks compared to standard acupuncture[37]*

**Reviewer #3:**

**The study is designed as a randomized controlled trial aiming to evaluate the efficacy and safety of Jin’s Three-Needle acupuncture therapy in patients with mild-to-moderate depression, while also exploring potential molecular mechanisms such as DNA methylation. The overall study design is relatively reasonable; however, the following questions need to be considered before further consideration.**

**1. Background : The background only discusses depression-related genes and inflammatory markers in relation to depression, but does not explain how acupuncture—particularly Jin’s Three-Needle—may be connected to these molecular mechanisms. The logical link is incomplete.**

We sincerely appreciate your insightful feedback on clarifying the mechanistic link between Jin’s Three-Needle (JTN) acupuncture and molecular pathways implicated in depression. we have revised the Background section to explicitly connect acupuncture effects to genetic and inflammatory mechanisms as follow:

*Recent studies have demonstrated that peripheral blood leukocytes from patients with depression exhibit elevated expression levels of depression-related genes, particularly inflammatory markers, which often display DNA methylation patterns resembling those observed in brain tissues[13, 14]. For instance, methylation of the glucocorticoid receptor gene NR3C1 and the serotonin transporter gene SLC6A4 in leukocytes has been linked to depressive symptoms[15, 16]. DNA methylation in peripheral blood leukocytes represents a promising biomarker for depression, as it frequently suppresses or blocks the transcription of genes associated with depressive symptoms[17, 18]. Research has indicated that acupuncture can reduce the production of pro-inflammatory cytokines[19]. Previous studies found that acupuncture treatment significantly decreased the serum levels of IL - 6 and TNF - α in patients with major depressive disorder. Acupuncture may suppress the activation of the nuclear factor - kappa B (NF-κB) signaling pathway in the brain of rats with depression - like behavior. The NF-κB pathway is a key regulator of inflammatory responses, and its inhibition by acupuncture may lead to a reduction in the production of inflammatory cytokines, thus exerting an anti-depressive effect[20]. We hypothesize that JTN treatment may modulate aberrant gene expression and DNA methylation patterns in the peripheral blood leukocytes of individuals with depression, potentially resulting in improved depressive symptoms.*

**2. Primary outcome: The protocol should clearly specify the comparison time point for the primary outcome (e.g., week 8).**

We have revised the 'Outcome' section to state clearly:

*"The primary outcome is the between-group difference in the mean change of the HAMD-17 total score from baseline to Week 8."*

The assessments at Weeks 4 and 6 will be analyzed as secondary time points to explore the trajectory and time-course of the therapeutic response using repeated-measures models.

**At present, all outcome measures are subjective scales, with no objective or semi-objective indicators to strengthen the robustness of the findings.**

We understand your concern that relying solely on subjective scales may limit the robustness of our findings. However, we have encountered several challenges that prevent us from incorporating objective or semi-objective indicators at this stage, as detailed below: Conducting objective or semi - objective measurements such as brain imaging (e.g., functional magnetic resonance imaging, fMRI) or biomarker analysis (e.g., measuring inflammatory cytokines) requires significant financial resources. Our current research budget is mainly allocated to essential aspects such as participant recruitment, acupuncture treatment materials, and compensation for participants and research staff. As a result, we are unable to allocate sufficient funds to cover the expenses associated with these objective or semi - objective measurements. However, the primary and overarching goal of this initial RCT is to establish the clinical efficacy of Jin's Three-Needle Acupuncture versus a sham control for mild-to-moderate depression. The field of acupuncture research urgently requires high-quality, methodologically rigorous trials that definitively answer the question of whether the intervention works from a clinical perspective. The HAMD-17, while subjective, is the gold standard and regulatory-accepted primary endpoint for antidepressant trials.

While we cannot add objective biomarkers, we have implemented several design features to maximize the robustness and validity of our findings based on subjective scales:

a. Use of a Validated Sham Control: The use of a sham acupuncture procedure is the most critical feature for controlling for non-specific effects (e.g., placebo, patient expectation, and therapist attention). Any significant difference in HAMD-17 reduction between the JTN and sham groups can be more confidently attributed to the specific physiological effects of the verum acupuncture.
b. Blinded Outcome Assessors: The clinicians administering the HAMD-17 will be blinded to the participants' group allocation, minimizing assessment bias.
c. Multiple Validated Scales (Convergent Validity): We employ a battery of secondary subjective scales (SDS, HAMA, SAS, PSQI). If a consistent, positive signal is observed across all these scales in favor of the JTN group, it significantly strengthens the conclusion that the treatment has a broad and genuine clinical effect, thereby reducing the likelihood that the finding is an artifact of a single scale.

**3. Sample size calculation: The calculation cites a prior study based on 6-week HAMD-17 changes and SD values, yet the protocol does not clarify the definitive primary comparison time point (6 or 8 weeks).**

We sincerely thank you for their meticulous attention to this detail. Upon double-checking the cited reference *[Zhao F-Y, Zheng Z, Fu Q-Q, Conduit R, Xu H, Wang H-R, Huang Y-L, Jiang T, Zhang W-J, Kennedy GA: Acupuncture for comorbid depression and insomnia in perimenopause: A feasibility patient-assessor-blinded, randomized, and sham-controlled clinical trial. Frontiers In Public Health 2023, 11:1120567.]*, we must correct our previous statement: it was our error. The data from the reference used for the sample size calculation were, in fact, based on HAMD-17 changes after an 8-week treatment period, not a 6-week period.

**Moreover, the minimal clinically important difference (MCID) or clinically acceptable effect size is not reported. The SD value used is unusually small compared to typical clinical studies, and its source and plausibility need to be verified.**

We acknowledge that ideally, the sample size calculation should be based on a well-established minimum clinically important difference (MCID) for the HAMD - 17 scale. However, as you are aware, currently, there is no universally agreed - upon and fixed MCID value for the HAMD - 17. Different studies have employed various methods to estimate the MCID, and the results have shown variations.

The HAMD - 17 is primarily used to assess the severity of depressive symptoms and evaluate treatment effects. The change in its total score can reflect the evolution of the disease condition. But determining a specific MCID value requires consideration of the specific research background and the estimation method used.

In our study, we referred to the difference found in a previous study as a preliminary reference for sample size calculation. We understand that this may not fully represent a clinically relevant difference. To address this issue, we plan to conduct a sensitivity analysis after the study is completed. This analysis will assess how different assumed MCID values (within a reasonable range based on existing literature and expert opinions) would affect the study's results and conclusions.

Moreover, during the study design phase, we consulted with several experts in the field of depression research and clinical practice. They suggested that, given the current lack of a definite MCID for the HAMD - 17, using the data from a relevant prior study as a starting point for sample size estimation is a practical approach. At the same time, we will ensure that the study has sufficient power to detect meaningful differences in treatment effects, even if these differences do not precisely match a pre - defined MCID.

We appreciate your attention to this important aspect of our study, and we are committed to ensuring that our research is methodologically sound and clinically relevant. We will continue to monitor the development of MCID research for the HAMD - 17 and incorporate any new findings into our future work.

We will clearly state these planned analyses in our updated statistical analysis plan.

**4. Safety considerations: Since enrolled patients are not receiving antidepressant medication, the potential safety risks associated with mood fluctuations during the intervention period are not adequately addressed.**

We appreciate this opportunity to detail the robust safety protocols we have implemented to address this very issue, which we will now describe more explicitly in the manuscript as follow:

*“All acupuncturists and research assessors will be trained in recognizing signs of clinical deterioration and are aware of the emergency referral procedures. Participants who experience significant worsening of their depression will be offered, in consultation with their treating physician, the option to initiate standard antidepressant medication. This would be considered a major protocol deviation and lead to study withdrawal, but their data would continue to be followed for safety analyses.”*

In summary, while the study design excludes concomitant antidepressants, it does not leave participants without a safety net. We have implemented a rigorous, proactive, and ethically sound safety monitoring plan to ensure that any participant experiencing mood fluctuations or clinical deterioration is promptly identified, assessed, and provided with appropriate clinical management, which may include psychiatric referral and initiation of standard care.

**5. Sham acupuncture design: The sham point locations are vague, and some are too close to active acupoints (e.g., 1 cun beside Sanyinjiao is Fuliu, which still has therapeutic effects). In addition, shallow needling may still induce physiological effects, compromising the credibility of the control condition.**

We have carefully considered your concerns regarding the vagueness of sham point locations and the potential therapeutic effects of shallow needling, and have made the following comprehensive revisions to enhance the credibility of the control condition:

We have re-evaluated and optimized the selection of sham acupoints. Instead of using a 1 - cun distance from the verum acupoints as the criterion for locating sham acupoints, we conducted an extensive review of classical acupuncture literature and modern acupuncture research. Based on this review, we identified non - meridian regions that are far from the verum acupoints and have no meridian associations as the new sham acupoint locations. The location of the Sanyinjiao is on the tibial aspect of the leg, posterior to the medial border of the tibia, 3 cun superior to the prominence of the medial malleolus. The Fuliu is on the medial side of the lower leg, directly above the Taixi point by 2 cun. Therefore, one cun lateral to Sanyinjiao has no acupoint. Detailed information on the location of these optimized sham acupoints can be found in Supplementary file S3.

Additionally, these specific non-acupoint locations have been successfully utilized as inert controls in previous high-impact acupuncture RCTs (e.g., studies published in JAMA and Annals of Internal Medicine: *[ Wu XK, Stener-Victorin E, Kuang HY, Ma HL, Gao JS, Xie LZ, Hou LH, Hu ZX, Shao XG, Ge J, Zhang JF, Xue HY, Xu XF, Liang RN, Ma HX, Yang HW, Li WL, Huang DM, Sun Y, Hao CF, Du SM, Yang ZW, Wang X, Yan Y, Chen XH, Fu P, Ding CF, Gao YQ, Zhou ZM, Wang CC, Wu TX, Liu JP, Ng EHY, Legro RS, Zhang H; PCOSAct Study Group. Effect of Acupuncture and Clomiphene in Chinese Women With Polycystic Ovary Syndrome: A Randomized Clinical Trial. JAMA. 2017 Jun 27;317(24):2502-2514. doi: 10.1001/jama.2017.7217. PMID: 28655015; PMCID: PMC5815063.]*). They are sufficiently distant from any verum acupoints to eliminate the risk of eliciting specific therapeutic effects.

**6. Blinding: The blinding strategy is insufficient. No blinding validation is planned, leaving uncertainty as to whether participants and assessors will remain effectively blinded.**

Following your suggestion, we have revised it as follow:

*“Acupuncturists will not be blinded to group allocation. However, the two groups of participants will avoid getting communicating with each other after randomization, and will receive treatment in separate treatment rooms to minimize subjective effects. The outcome assessors, data collectors, and statisticians will be blinded to group allocations. An independent researcher will supervise the conversation between participants and acupuncturists to avoid them communicating issues related to the allocation. At mid-study and end-study, participants will be asked via anonymous questionnaires to guess their assigned group and provide reasons. Statistical analysis of the results will be used to check whether participants remained blinded. If a significant number guess correctly, potential causes will be analyzed and measures will be adjusted. After each assessment, assessors will reassess the group allocations of randomly selected cases without access to their prior assessment results, while their decision-making processes will be recorded. The comparison between initial and repeated judgments will be used to assess whether assessors will be influenced.”*

**7. Molecular testing details: The protocol lacks essential methodological details for blood sample testing, including sampling time points, tube type, processing window, storage conditions (−80°C?), DNA extraction method and QC standards, as well as the specific testing platform and analysis strategy.**

We have carefully considered your comments and made comprehensive revisions to address the lack of essential methodological details. Details on molecular analysis are provided in S4 of the Supplementary File as follow:

*“Collection of patient blood samples*

*Whole blood will be collected from 8 patients before and after intervention, and from 10 healthy volunteers. Draw 10ml of venous blood on an EDTA anticoagulant tube on an empty stomach. Let it stand at room temperature for 1 hour. Then, centrifuge at 3000rpm for 10 minutes at 4℃ to obtain the supernatant plasma sample. DNA will be isolated from the peripheral blood leukocytes using QIAamp DNA mini kit (Qiagen) . The isolated DNA will be stored at - 80°C, with a temperature monitoring system in place to maintain the required storage conditions.*

*Data quality control and standardization*

*The Illumina iScan system scans the chip to generate ldat files. These ldat files are imported into R, and the ChAMP package will be used for preprocessing to obtain the raw data. The raw data (idat files) will be normalized using the BMIQ method. Signal values (Beta values) representing DNA methylation levels at CpG sites will be then extracted. Beta values (β) range from 0 to1, indicating the methylation level at a given site. Values closer to 0 suggest lower methylation, while values closer to 1 indicate higher methylation. The calculation formula for the Beta value is as follows:*

*Betai=Mi/(Mi+Ui+offset)*

*"M" represents the methylation signal intensity, "U" represents the non-methylation signal intensity, and "offset" is an adjustment value used to prevent the denominator from being zero.*

*DNA methylation analysis*

*The MethylationEPIC BeadChip experiment and data analysis of all samples will be conducted by OE Biotechnology Co. Ltd. (Shanghai, China). DNA concentration and integrity will be evaluated by NanoDrop 2000 spectrophotometer (Thermo Fisher Scientific, Waltham, MA, USA) and agarose gel electrophoresis respectively. DNA will be treated with bisulfite using the Zymo Research EZ DNA methylation-Glod Kit (Zymo Research, Irvine, CA, USA), and analyzed on the Illumina Infinium MethylationEPIC (850 K) BeadChip (Illumina). The chips will be scanned with Illumina iSCAN, and the Idat files will be imported and preprocessed with ChAMP (version 2.12.4) package in R to obtain the raw data. The BMIQ method will be used to normalize the raw data.”*

**8. Inclusion/exclusion criteria: The upper age limit of 45 years is not justified, and common acupuncture contraindications (e.g., bleeding tendency, local infection, implanted electronic devices) are not listed in the exclusion criteria.**

We have carefully considered your comments and have revised it as follw:

*“Inclusion criteria*

*Participants who meet the following criteria will be included.*

*①Patients who fit the diagnostic criteria for depression in the Diagnostic and Statistical Manual of Mental Disorders(DSM-5);*

*② Patients with a HAMD-17score between 8 and 25.*

*③Patients between the ages of 18 and 65 years;*

*④Patients are voluntary and able to sign informed consent.*

*Exclusion criteria*

*① Patients having suicidal tendencies (HAMD third score greater than 2 points), bipolar depression, and treatment-resistant depressions;*

*②Patients having immediate family or personal history of mental illness;*

*③Patients who have serious physical health (as assessed by a doctor);*

*④Patients who are pregnant or planning to become pregnant;*

*⑤ Patients who have taken antidepressants or receive acupuncture treatment within three months.*

*⑥Patients with contraindications to acupuncture, including: a. Coagulation disorders (e.g., hemophilia) or current use of anticoagulant medication (e.g., warfarin); b. Severe thrombocytopenia; c. Local skin infections, ulcers, or scars at proposed needling sites; d. Presence of an implanted electronic medical device (e.g., pacemaker, spinal cord stimulator); e. Known severe cardiovascular conditions (e.g., uncontrolled arrhythmia).”*

**9. There are spelling mistakes in the names of scales such as SAS/HAMA (e.g., line 247). Consistent proofreading across the manuscript is needed.**

We have thoroughly proofread the entire manuscript to correct all spelling, typographical, and formatting errors.

**10. The authors describe inflammatory markers and related assays as “mechanistic exploration,” but in other sections they categorize them as efficacy/safety outcomes. This is inconsistent and confusing.**

Following your suggestion, we have completely removed all references to inflammatory markers and related assays from the efficacy/safety outcomes section. The detailed description of the mechanistic exploration involving these markers and assays has been relocated to the statistical analysis section.

**11.** **No post-intervention follow-up period is planned, making it impossible to evaluate the durability and long-term effects of treatment.**

We thank the reviewer for this important comment regarding the long-term durability of the treatment effect. We fully acknowledge that the absence of a post-intervention follow-up period is a limitation of our study, and we will explicitly state this in the manuscript's discussion section. We would like to explain the reasons why we are unable to arrange a follow - up period after treatment in this study. Firstly, our current research budget is strictly limited, and allocating funds for an extended follow - up would severely impact our ability to carry out the core aspects of the study, including the initial treatment phase and the immediate post - treatment data collection. Secondly, a major obstacle is the difficulty in ensuring participant retention over an extended follow - up period. In our study population, many participants have busy schedules, and some may move away from the study area during the follow - up period. Previous research in similar settings has shown that participant drop - out rates can be quite high in long - term follow - up studies, especially when there is no strong incentive for participants to continue participating.
